# Effect of intraoperative intravenous remimazolam on the postoperative quality of recovery after noncardiac surgery: A meta-analysis of randomized controlled trials

**DOI:** 10.1371/journal.pone.0319044

**Published:** 2025-03-20

**Authors:** Huan Liu, Mingxia Zhou, Xiangdong Chen, Elham Esmaeili, Meng Sun, Zhilin Wu

**Affiliations:** 1 Department of Anesthesiology, Union Hospital, Tongji Medical College, Huazhong University of Science and Technology, Wuhan, China; 2 Institute of Anesthesia and Critical Care Medicine, Union Hospital, Tongji Medical College, Huazhong University of Science and Technology, Wuhan, China; 3 Key Laboratory of Anesthesiology and Resuscitation (Huazhong University of Science and Technology), Ministry of Education, China; 4 Operation room, Xinzhou district people’s hospital, Wuhan, China; The Fourth Affiliated Hospital Zhejiang University School of Medicine, CHINA

## Abstract

**Objectives:**

The postoperative quality of recovery holds significant economic and prognostic implications. Numerous studies have investigated the postoperative quality of recovery following surgery. However, the impact of intraoperative intravenous remimazolam on postoperative recovery has yielded conflicting results.

**Methods:**

In this qualitative review of randomized controlled clinical trials assessing the use of intraoperative remimazolam, we present the effects of intravenous remimazolam on postoperative recovery quality in noncardiac surgery patients. We conducted a comprehensive search of PubMed, MEDLINE, Web of Science and Cochrane Library for randomized controlled trials published up to September 23, 2024, without any language restrictions, to investigate the impact of intraoperative intravenous remimazolam on the quality of postoperative recovery following noncardiac surgery. The primary outcome measure was the quality of postoperative recovery assessed using global QoR-40 scores or QoR-15 scores. Secondary outcomes included five dimensions of quality of postoperative recovery: physical comfort, emotional state, physical independence, psychological support, and pain. As well as time to extubation, VAS score, PACU stay, and days in hospital. We analyzed the pooled data using a random-effects model.

**Results:**

This meta-analysis included 18 studies published between 2022 and 2024 involving 1720 patients. The quality of postoperative recovery was assessed using the QoR-15 or QoR-40 questionnaires. The pooled results showed no significant difference in QoR scores between the two groups on the first postoperative day (SMD =  0.12; 95% CI -0.13-0.36; I^2^ = 78%; p =  0.35). Furthermore, differences in QoR dimensions, PACU duration, extubation time, and length of hospital stay were not observed.

**Conclusion:**

Our analysis showed that the quality of recovery after general anaesthesia in the remimazolam group was similar to that of conventional sedation. Therefore, remimazolam may be used as a potential anaesthetic agent as an alternative to conventional sedation for non-cardiac surgical anaesthesia.

## Introduction

With the significant advancements in surgical techniques, anesthesia, sedation, and pain management, there is now more emphasis on the quality of perioperative recovery beyond morbidity endpoints, as it is closely related to patient subjective well-being and satisfaction [1,2]. The Quality of Rehabilitation (QoR) score is a patient-centered objective measure that evaluates the overall health of a patient following surgery and anesthesia [3]. The QoR-15 and QoR-40 scales are widely used to assess the quality of recovery after surgery. The QoR-40 is the most comprehensive global measure of a patient’s perioperative health and includes the following five dimensions: physical comfort, emotional state, physical independence, psychological support, and pain [4,5]. The QoR-15, on the other hand, is a compendium of the strongest psychometric questions for each of these five dimensions.The QoR-40 scores range from 40–200, and the scores for the QoR-15 range from 0–150, and both rate health status as the higher the score the better the quality of recovery. These scales have been validated in patients undergoing various surgical procedures and are commonly used in clinical assessments of postoperative recovery quality [6].

Propofol and inhalation anesthesia are commonly used in general anesthesia to maintain sedation. However, according to studies, total intravenous anesthesia (TIVA) results in better quality of recovery than inhalation anesthesia [7]. Remimazolam is a recently developed benzodiazepine with ultra-short action properties. It presents several advantages, such as rapid onset and offset, metabolism induced mostly by tissue esterase, inactive metabolites, independence from liver and kidney function, high hemodynamic stability, and the possibility of being antagonized by flumazenil [8]. A multitude of clinical trials have showcased its safety and efficacy during induction and maintenance of general anesthesia, in comparison with propofol [9,10].

Currently, numerous studies examine the impact of intraoperative intravenous remimazolam during general anesthesia on postoperative recovery quality. However, these studies feature small, single-center samples, and their results are conflicting. Therefore, the objective of this meta-analysis is to assess the effect of intravenous remimazolam on the quality of postoperative recovery for non-cardiac surgery patients experiencing general anesthesia.

## Methods

### Search strategy and selection criteria

We performed a systematic review and meta-analysis of clinical trials examining the impact of intraoperative intravenous remimazolam on postoperative recovery quality in adult patients undergoing noncardiac surgery under general anesthesia. This meta-analysis conforms to the Preferred Reporting Items for Systematic Reviews and Meta-Analyses (PRISMA) Statement (S5 File) and was registered with the International Prospective Register of Systematic Reviews (CRD42023475274) [11]. We searched PubMed, EMBASE, Cochrane and the Web of Science databases for relevant studies published until September 23, 2024. No language restrictions were applied. The detailed search strategy is available for reference in S1 File. We assessed all potentially suitable studies for review, regardless of language or primary outcome (S2 File and S3 File). Additionally, we performed a manual search of the references of the included studies to identify any original articles that were not previously retrieved.

### Data extraction and endpoints

The inclusion criteria for this article were as follows (1) studies of people aged > 18 years undergoing general anaesthesia; (2) randomized controlled trials of general anaesthesia maintenance using remimazolam as an intervention and isoproterenol, inhalational anaesthetics, and placebo as a control; and (3) studies that included at least one of the following outcome metrics: QoR scores, visual analogue scale (VAS) scores, time to extubation, post-anesthesia care unit (PACU) stay, and length of hospital stay. Exclusion criteria were (1) duplicate articles, (2) studies involving patients undergoing cardiac surgery, and (3) studies that contained inadequate data for meta-analysis. Two independent investigators conducted a preliminary assessment of the titles/abstracts and extracted the data. After selecting articles based on the inclusion and exclusion criteria, each article was read in full-text. If there was any disagreement, a third investigator acted as the final arbiter. Data were recorded using a standardized form prepared by the authors, which collected information on patient age, anesthesia process, remimazolam dosage and administration method, procedures performed, and outcomes. The primary outcome of the study was evaluated using the QoR-15 scale and/or the QoR-40 questionnaire, focusing on postoperative quality of recovery. Secondary outcomes measured included postoperative emotional state, pain, physical comfort, physical independence, and psychological support as well as time to extubation, duration of hospital stay, and PACU stay.

### Quality assessment

The risk of bias was independently assessed by two reviewers using the Cochrane Handbook’s tool for RCT bias. They evaluated the adequacy of randomization, allocation concealment, blinding, completeness of outcome data, selective reporting, and other potential sources of bias. The quality of evidence for the primary outcomes was appraised using the Grading of Recommendations Assessment, Development, and Evaluation (GRADE) methodology, and a summary of findings was generated via GRADEpro software. The detailed GRADE assessment for each outcome is available for reference in S4 File. In cases where discrepancies arose between the two reviewers, a third reviewer intervened to mediate discussions until consensus was achieved.

### Statistical analysis

We performed a sensitivity analysis to investigate the sources of heterogeneity across the studies. To calculate the statistical heterogeneity, we used the chi-square test (χ²) and the Higgins test (I²) [12]. The presence of heterogeneity was established at p <  0.05 and I² ≥  50%. For dichotomous variables, we estimated the Odds Ratio (OR) with a 95% Confidence Interval (CI), while for continuous variables, we reported the Mean Difference (MD) to assess the statistical difference between groups. Although QoR-40 scores and QoR-15 scores are two distinct scales to ascertain postoperative recovery quality, we adopt the standardized mean difference (SMD) for the forest mapping. After performing qualitative analysis of the studies and statistical heterogeneity assessment, we utilized the DerSimonian-Laird 20 method to implement the random effects model [13]. Additionally, we evaluated potential publication bias through visual analysis of the funnel plot [14,15]. Additionally, we conducted a sensitivity analysis to assess the stability and reliability of the pooled results. All analyses were performed utilizing Review Manager (RevMan) software version 5.3, developed by the Cochrane Collaboration in Copenhagen, Denmark. Statistical significance was set at <  0.05 for all two-tailed P values.

## Results

**Fig 1:**
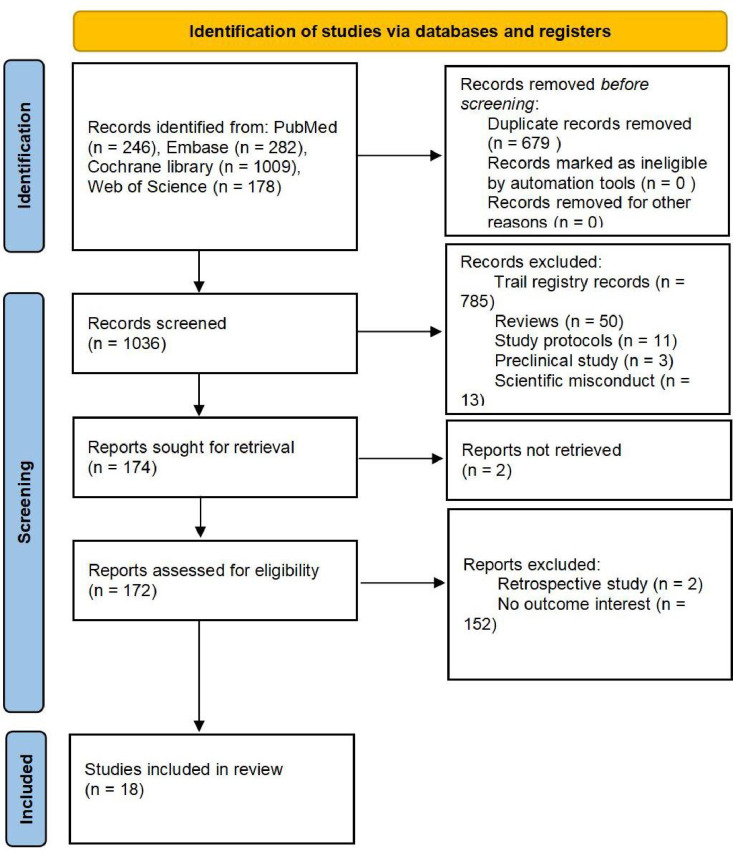
Study selection process.

Fig 1 illustrates the identification of 1715 studies, including 246 on PubMed, 282 on Embase, 1009 on the Cochrane library, and 178 on the Web of Science. From these studies, eighteen were selected to constitute this meta-analysis. Table 1 provides details on the characteristics of the eighteen trials selected eleven studies were conducted in China [16–26], and seven were carried out in Korea [27–33].

**Table 1:** Characteristics of the studies included in the meta-analysis.

	Country	Mean age cases/controls	Number cases/controls	Type of surgery	Trial interventions(induction, maintenance) cases/controls	Follow-up: time of patients, cases/controls
Mao et al., 2022	China	52.5 ± 17.5/ 50.0 ± 25.8	64/64	urologic surgery	remimazolam (0.2–0.3) mg/kg, remimazolam(1–2)mg/kg/h; propofol (2–3) mg/kg, propofol (4–10) mg/kg/h.	POD1 and POD3
Choi,et al., 2022	Korea	39.5 [33–48]/ 41.0 [37–47]	70/69	Partial thyroidectomy; Total thyroidectomy; Thyroidectomy combined with RND	remimazolam 6 mg/kg/h, remimazolam(1–2)mg/kg/h; propofol infusion (TCI) with an effect-site concentration (Ce) of 5 μg/mL and 2–6 μg/mL for anesthesia induction and maintenance, respectively.	POD1 and POD2
Song et al., 2022	Korea	43.4 ± 10.4/ 43.2 ± 9.0	82/83	Laparoscopic cholecystectomy; Robotic gynecologic surgery	propofol 2 mg/kg, remimazolam1-2 mg/kg/h; propofol 2 mg/kg, 0.7-0.9 MAC of desflurane.	1day before and after surgery
Kim et al., 2023	Korea	41.7 ± 12.2/ 43.3 ± 13.2	94/95	Cyst enucleation; Tooth extraction; Tumor resection	remimazolam 12 mg/kg/h, remimazolam (1–2) mg/kg/h; induced and maintained with propofol (target effect-site concentration: 3–5 µg/ml).	POD1
Zhao et al., 2023	China	65.4 ± 3.1/ 64.5 ± 3.0	54/54	Thoracoscopic laparoscopic radical esophagectomy	remimazolam 0.2-0.3 mg/kg, remimazolam0.4-1.0 mg/kg/h; propofol1‑2 mg/kg, propofol 4‑10 mg/kg/h.	1 day before surgery; POD1 and POD3
Chen et al., 2024	China	28.5[23–33.2]/ 32.0 [28–34]	54/54	Laparoscopic sleeve gastrectomy	remimazolam 0.2-0.3 mg/kg, remimazolam 0-1.0 mg/kg/h and 0.7 MAC of sevoflurance; propofol 1.5‑3 mg/kg, propofol 0‑12 mg/kg/h and 0.7 MAC of sevoflurance.	POD1
Deng et al., 2023	China	70.8 ± 4.4/ 71.8 ± 5.5	54/54	total joint arthroplasty under neuraxial anesthesia	remimazolam 0.025–0.1 mg/kg, remimazolam 0.1–1.0 mg/kg/h; dexmedetomidine 0.2–0.7 μg/kg/h.	POD1, POD2 and POD3
Gao et al., 2023	China	59.9 ± 7.3/ 58.9 ± 10.8	30/30	bronchoscopy under intravenous anesthesia	remimazolam 6 mg/kg/h, remimazolam 0.6–2 mg/kg/h; propofol 2 mg/kg, propofol 4-6 mg/kg/h.	1day before and after surgery
Huang et al., 2023	China	62.6 ± 8.9/ 63.8 ± 11.0	60/60	Breast cancer surgery	Remimazolam 0.3 mg/kg, remimazolam0.3 mg/kg/h and 0.5-1 MAC of sevoflurance; propofol 2 mg/kg, propofol 2 mg/kg/h and 0.5-1 MAC of sevoflurance.	POD1
Lee et al., 2024	Korea	49.1 [35–67]/ 49.2 [30–68]	36/36	Anterior cervical discectomy and fusion (ACDF)	remimazolam infusion 6–12 mg/kg/h, maintained with remimazolam(1–2)mg/kg/h; propofol (1–2) mg/kg, sevoflurane inhalation at 1.5–2 vol%.	POD1 and POD2
Lee et al., 2024	Korea	54.2 [27–66]/ 50.3 [33–66]	36/36	Spine surgery	remimazolam infusion 6–12 mg/kg/h, remimazolam(1–2)mg/kg/h; targeted controlled infusion (TCI) of propofol set at 3.0 ng mL − 1.	POD1
Lee et al., 2023	Korea	45 ± 13.4/ 51 ± 12.1	28/29	elective open thyroidectomy	remimazolam 6mg/kg/h until the patient was unconscious, remimazolam(1–2)mg/kg/h; target-controlled infusion (TCI) of propofol set at 3.0 ng.ml − 1, a minimum of 2.0 ng.ml − 1.	POD1
liao et al., 2023	China	70.1 ± 3.5/ 71.2 ± 3.58	34/35	Laparoscopic radical resection of gastric cancer	propofol 1.0–1.5mg/kg and remimazolam 0.2mg/kg, propofol 4–8 mg/kg/h; dexmedetomidine 0.5μg/kg over 10min and intravenous propofol 1.0–1.5mg/kg, propofol 4–8 mg/kg/h.	POD3 and POD7
luo et al., 2023	China	70.1 ± 3.5/ 71.2 ± 3.58	38/38	general anesthesia included: Urology, Obstetrics and Gynecology, General surgery, Thoracic surgery	remimazolam 0.3mg/kg, remimazolam 1–3mg/kg/h; propofol 2.0–2.5mg/kg, propofol 6–12 mg/kg/h.	POD1 and POD14
Ryu et al., 2024	Korea	71.7 ± 9.4/ 66.1 ± 11.9	17/17	transurethral resection of bladder tumor(TURBT)	remimazolam 12 mg/kg/h, remimazolam targeting PSI of 30 to 50; propofol 1.5–2 mg/kg and sevoflurane 5 vol%, sevoflurane 1.5–2 vol%.	1 day before and after surgery
Tang et al., 2023	China	48.5 [19–62]/ 50 [19–64]	56/58	ambulatory arthroscopic meniscus repair	remimazolam6 mg/kg/h, remimazolam 0.4-2mg/kg/h; targeted controlled infusion (TCI) of propofol to 1-3 ugmL-1.	POD1
Xin et al., 2024	China	81.5 ± 4.9/ 82.3 ± 6.0	53/55	endoscopic retrograde cholangiopancreatography (ERCP)	remimazolam 0.15–0.2 mg/kg, remimazolam 0.4-0.8 mg/kg/h; propofol 1–1.5 mg/kg, propofol 2-6 mg/kg/h.	POD1 and POD3
Zhang et al., 2024	China	49 ± 9.4/ 47.6 ± 10.1	65/63	Laparoscopic-cholecystectomy; Internal fixation removal	remimazolam0.3 mg/kg, remimazolam 1-1.5 mg/kg/h; propofol 2–2.5 mg/kg, propofol 4-8 mg/kg/h.	POD1

POD1: postoperative day 1; POD2: postoperative day 2; POD3: postoperative day 3; POD7: postoperative day 7; POD14: postoperative day 14

**Fig 2:**
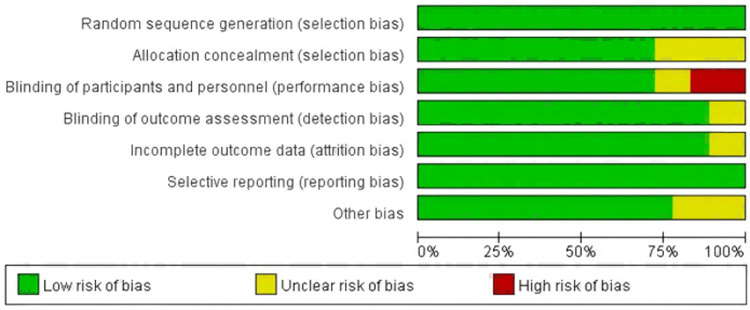
Bias risk of the eligible studies.

**Fig 3:**
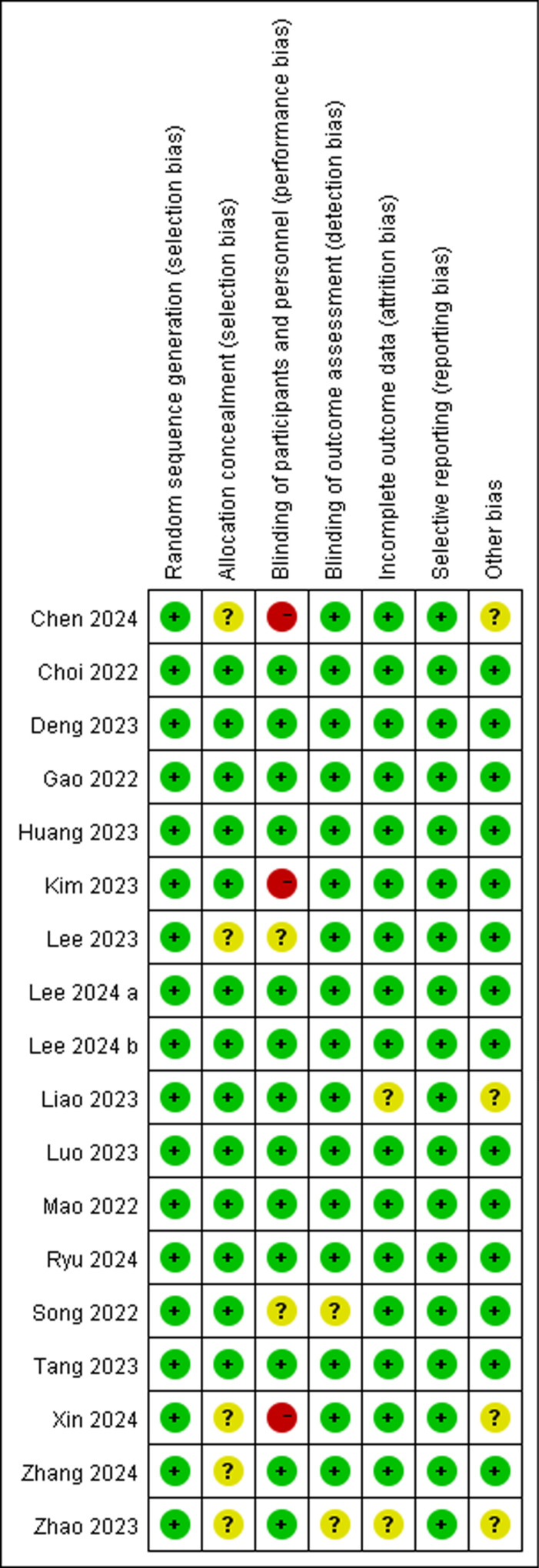
Sensitivity and specificity of the included studies. “+”represents low risk of bias; “?” stands for unclear risk of bias;“-” represents high risk of bias.

The risk of bias graphs and summaries for each study are presented in Figs 2 and 3. The included studies demonstrated relatively low levels of methodological bias, indicating high quality. Among the eighteen randomized controlled trials (RCTs), five RCT did not report the method of allocation concealment, leading to an unclear risk of bias. Two trials showed an unclear risk of bias in blinding of participants and personnel, while three trial demonstrated a high risk in the same domain. Two trials demonstrated unclear risk of bias in blinding of outcome assessment. Moreover, two trial exhibited an unclear risk of bias in incomplete outcome data.

**Fig 4:**
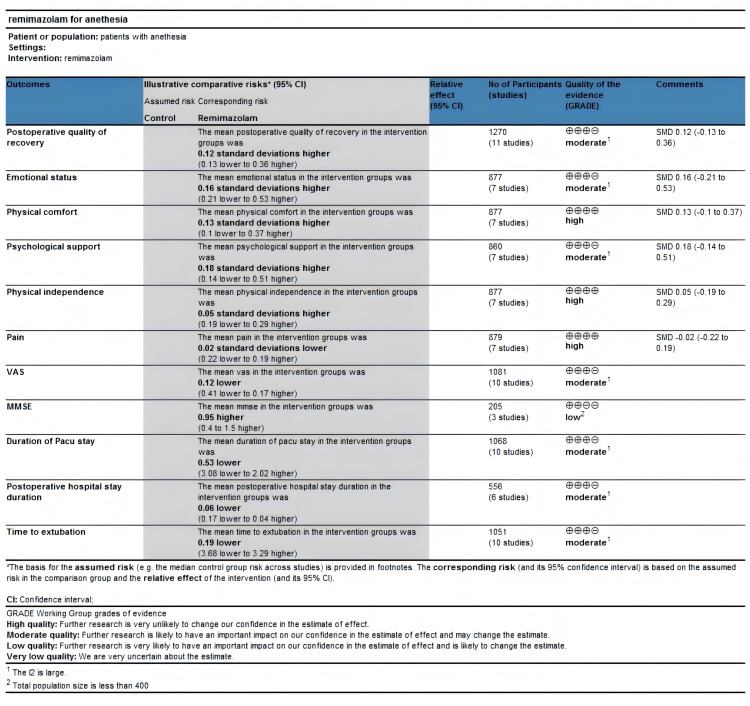
Grading of Recommendations Assessment, Development and Evaluation (GRADE) summary of findings table.

**Fig 5:**
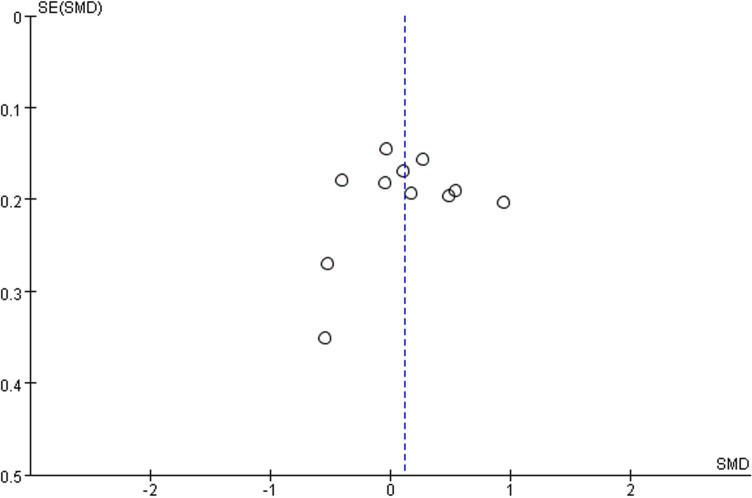
Funnel plot of publication bias.

The GRADE summary of findings table is shown in Fig 4. Seven outcomes were deemed to have insufficient consistency due to high heterogeneity. One outcome was considered to have insufficient precision due to a limited sample size. As for publication bias, a visual inspection of the funnel plot revealed no significant evidence of publication bias (Fig 5).

**Fig 6:**
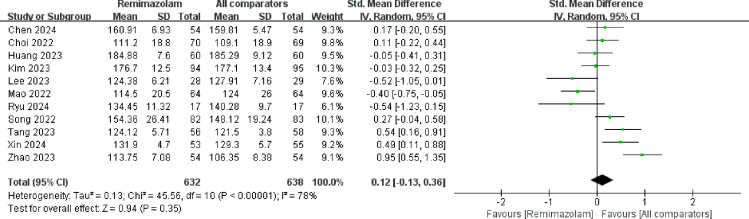
Meta-analysis of remimazolam group on the postoperative quality of recovery after noncardiac surgery. IV, weighted mean difference; df, Degrees of freedom.

**Fig 7:**
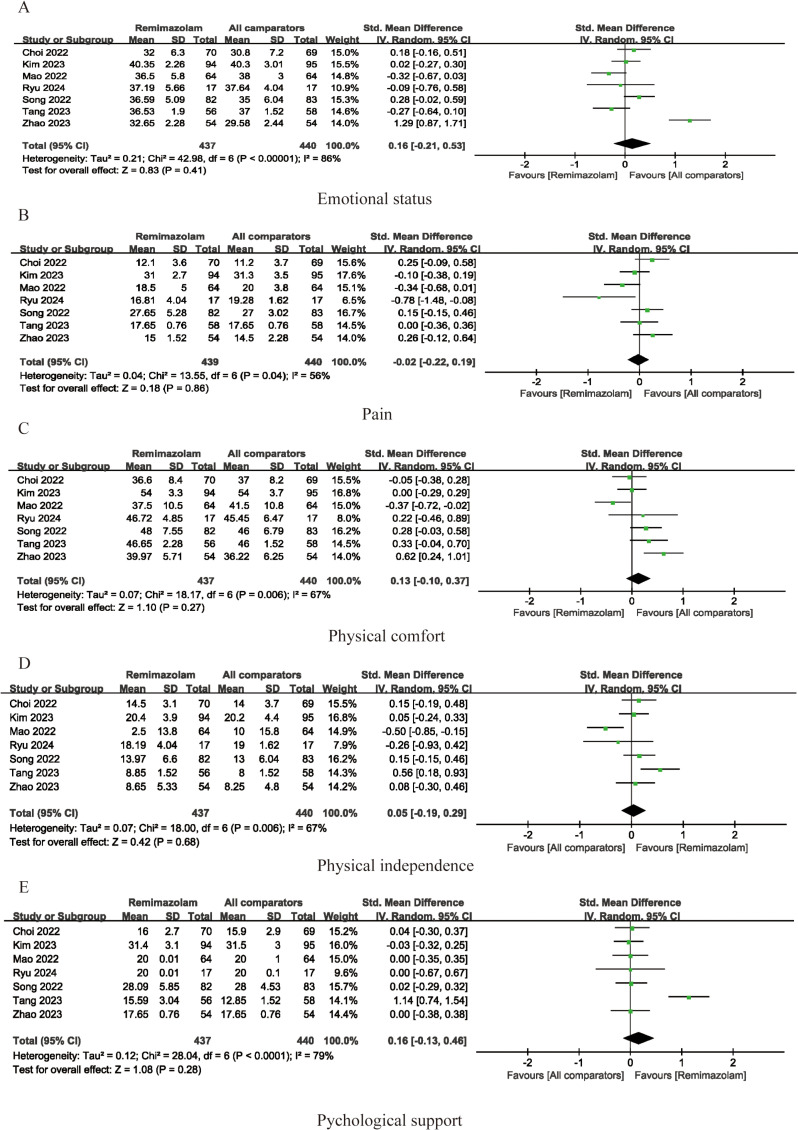
Forest plots of pooled studies for emotional status(A), pain(B), Physical comfort(C), Physical independence(D) and Psychological support(E). IV, weighted mean difference; df, Degrees of freedom.

**Fig 8:**
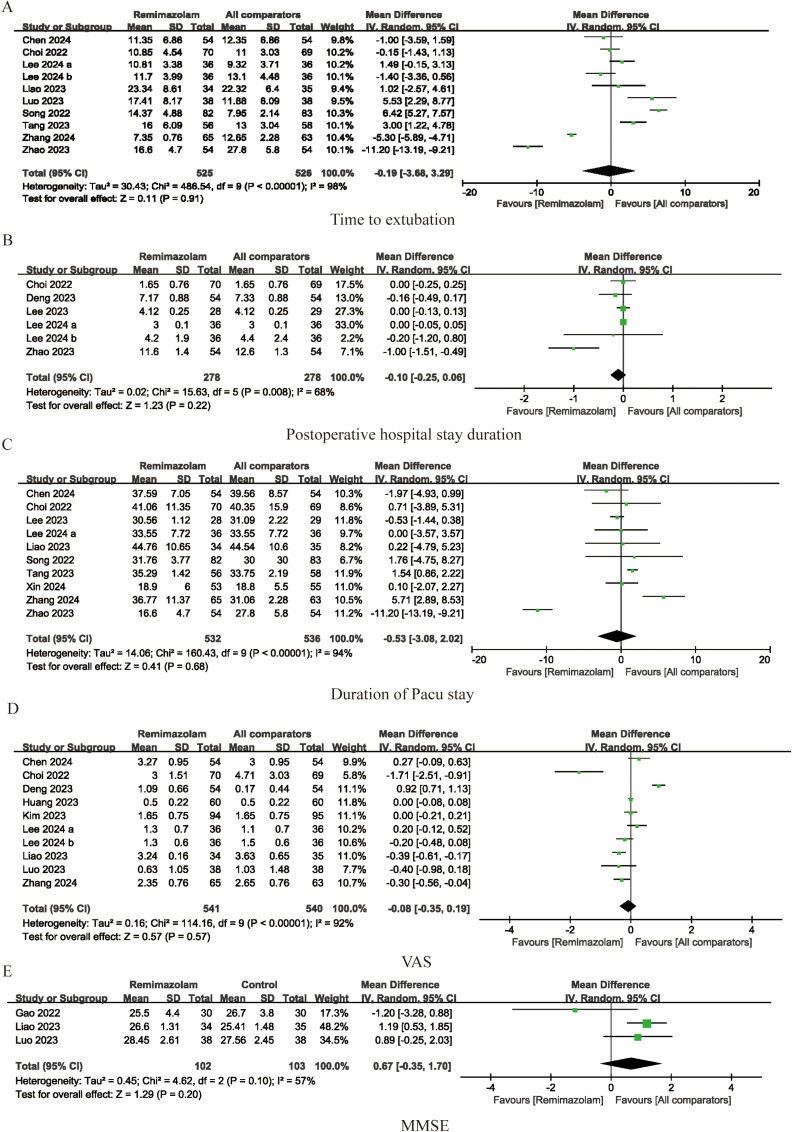
Forest plots of pooled studies for time to extubation (A), postoperative hospital stay duration (B), duration of pacu stay (C), VAS (D), and MMSE(E). IV, weighted mean difference; df, Degrees of freedom.

Postoperative quality of recovery was assessed in eleven studies, as illustrated in Fig 6. Our findings indicate that administration of remimazolam provides a similar quality of postoperative recovery as conventional sedation (SMD =  0.12; 95% CI -0.13-0.36; I^2^ = 78%; p =  0.35). Emotional status, pain, physical comfort, physical independence, and psychological support were evaluated separately in 7 studies, and meta-analysis did not uncover any significant differences between the remimazolam and placebo groups (emotional status SMD =  0. 16; 95% CI = -0.21-0.53; I^2^ = 86%; p =  0.41; SMD for pain =  -0.02; 95% CI -0.22-0.19; I^2^ = 56%; p =  0.86; SMD for physical comfort =  0.13; 95% CI -0.10-0. 37; I^2^ = 67%; p =  0.27; SMD for physical independence =  0.05; 95% CI =  -0.19-0.29; I^2^ = 67%; p =  0.68; SMD for psychological support =  0.16; 95% CI =  -0.13-0.46; I^2^ = 79%, p-value =  0.28, respectively). (See Fig 7). We also compared other postoperative recovery indicators, such as extubation time, PACU stay duration, postoperative hospital stay, postoperative VAS scores, and postoperative Mini-Mental State Examination (MMSE) scores. The results showed no differences between remimazolam and other anesthetic agents in these recovery indicators (time to extubation SMD =  -0. 19; 95% CI = -3.68-3.29; I^2^ = 98%; p =  0.91; SMD for postoperative hospital stay duration =  -0.10; 95% CI -0.25-0.06; I^2^ = 68%; p =  0.22; SMD for duration of PACU stay =  -0.53; 95% CI -3.08-2.02; I^2^ = 94%; p =  0.68; SMD for VAS =  -0.08; 95% CI =  -0.35-0.19; I^2^ = 92%; p =  0.57; SMD for MMSE =  0.67; 95% CI =  -0.35-1.70; I^2^ = 57%, p-value =  0.20, respectively) (See Fig 8).

In this study, to comprehensively assess the robustness of the meta-analysis results, we performed a sensitivity analysis using the leave-one-out method for all included outcomes. After removing any single study that could potentially affect result stability, the meta-analysis results remained unchanged, indicating that our pooled effect estimates exhibit strong robustness.

## Discussion

Surgery, anesthesia, quality of care, and patient factors affect postoperative recovery [34]. Currently, numerous studies are being conducted to identify an anesthetic method that enhances postoperative recovery quality [35]. This meta-meta-analysis examines the effect of intraoperative intravenous remimazolam on postoperative recovery quality based on a selected few studies. Results suggest that it is a promising intravenous anesthetic with potential advantages. The meta-analysis adhered to predetermined criteria and included eighteen randomized clinical trials that were published between 2022 and 2024. The objective was to evaluate the impact of intraoperative intravenous remimazolam on the quality of recovery during the postoperative period of adult patients who underwent general anesthesia. The combined analyses revealed no significant association between the remimazolam group and the control group (including propofol or desflurane) in terms of postoperative quality of recovery. All of the included studies were single-center randomized clinical trials with small sample sizes; therefore, the findings should be interpreted with caution.

Mao et al. found that remimazolam may lead to a temporary decrease in recovery quality compared to propofol for patients undergoing urologic surgery [16]. Remimazolam-based total intravenous anesthesia yielded comparable quality of recovery to propofol among female patients receiving thyroid surgery [27]. Administering remimazolam may serve as a viable option for improving recovery outcomes. Total intravenous anesthesia maintained with remimazolam provides superior recovery quality compared to inhalant anesthesia in patients undergoing laparoscopic surgery [33]. There were no significant intergroup differences in recovery quality between remimazolam and propofol groups during noninvasive oral and maxillofacial surgery [28]. However, patients with esophageal carcinoma in the remimazolam group had significantly higher Qor-15 scores than those in the propofol group [17]. Given these contradictory findings, it is necessary to conduct this meta-analysis, using quantitative methods to comprehensively assess the diversity of these studies.

Ultimately, our analysis indicates that remimazolam offers a recovery quality comparable to that of traditional sedatives. Postoperative recovery quality encompasses five domains: physical comfort, emotional state, physical independence, psychological support, and pain [36]. Across all five dimensions, there were no significant differences between the remimazolam group and the control group. Since immediate postoperative outcomes may not fully reflect the overall recovery, we further analyzed extubation time, VAS scores, PACU stay duration, and length of hospitalization to provide a more comprehensive evaluation of remimazolam’s impact on recovery quality. The results of these analyses showed no significant differences between the two groups.

Currently, most research on remimazolam focuses on procedural sedation. A meta-analysis revealed that remimazolam can lower the risk of bradycardia, hypotension, respiratory depression, and injection pain in patients undergoing procedural sedation, compared to propofol. However, there were no significant differences in sedation success rate, risk of postoperative nausea and vomiting (PONV), dizziness, time to loss of consciousness, recovery, and discharge between the two sedatives [37]. Studies on remimazolam remain controversial, but it is certain that it is a safe and effective procedural sedative. Remimazolam used for general anesthesia reduced the incidence of hypotension, hypoxemia, nausea, vomiting, dizziness, and injection site pain, thus indicating that it is a safer sedative [38]. While the benefits of remimazolam for postoperative recovery can be inferred based on this evidence, no definitive conclusion has been reached. Perhaps because remimazolam has not been extensively used in clinical practice and is still being studied, the author suggests the need for a larger sample to validate the findings.

This study utilized meta-analysis to evaluate the correlation between intraoperative intravenous remimazolam and postoperative quality of recovery after noncardiac surgeries. Our study has several strengths. We only included articles on intravenous remimazolam and limiting the review to RCTs. Postoperative quality of recovery was assessed in the included studies within one day after surgery, as this was the commonly used timepoint and most patients recovered within 2 days after surgery [39]. The Quality of Recovery-15 (QoR-15) or QoR-40 questionnaire was utilized to evaluate postoperative quality of recovery in all articles while minimizing the effects of publication and other biases.

Our study has several limitations. First, the included study population consists entirely of Asians, and we did not find any studies on remimazolam’s effect on postoperative recovery quality in non-Asian populations. Therefore, it remains unclear whether the conclusions of this meta-analysis are applicable to non-Asian groups. We searched the literature on remimazolam but did not come across studies focusing on racial subgroups. In a meta-analysis by Tang et al., which included six Chinese trials and five U.S. trials, no subgroup analysis by race was performed [40]. Similarly, Zhu et al. did not conduct such analysis [41]. Second, despite our efforts to address heterogeneity through strict inclusion and exclusion criteria, differences in anesthetic strategies, the dosage of remimazolam and propofol, and the types of surgery across studies increased heterogeneity in the results. Due to the limited number of studies with similar characteristics, we were unable to perform subgroup analyses based on dosage or surgery type. Third, the age ranges of patients varied across the studies, making it difficult to determine whether age influences recovery quality scores, which warrants further subgroup analysis in future research. Lastly, because the existing studies mainly present conflicting results regarding recovery quality on the first postoperative day and not all studies assessed recovery on the second or third day, our analysis focused solely on recovery quality on the first postoperative day.

## Conclusion

Our meta-analysis indicates that remimazolam provides comparable recovery outcomes to traditional sedatives in general anesthesia for non-cardiac surgeries. Additionally, there were no disadvantages in terms of postoperative pain, length of hospital stay, PACU duration, or extubation time. Given remimazolam’s hemodynamic superiority, it offers anesthesiologists more options when managing patients with hemodynamic instability. This study focused solely on early postoperative recovery, but the long-term effects should not be overlooked. Future research should focus on specific surgical populations and extend follow-up periods to assess long-term outcomes, contributing to the optimization of anesthesia management.

## Supporting information

S1 File Detailed search strategy.
(DOCX)

S2 File Raw data.
(XLSX)

S3 File Literature information retrieved.
(XLSX)

S4 File Detailed GRADE assessment for each outcome.
(DOCX)

S5 File PRISMA_2020_checklist.
(PDF)
