# Supplementary material for: Effect of intraoperative intravenous remimazolam on the postoperative quality of recovery after noncardiac surgery: A meta-analysis of randomized controlled trials
Source: PLoS One. 2025 Mar 20;20(3):e0319044. doi: 10.1371/journal.pone.0319044 (PMC11925310; doi:10.1371/journal.pone.0319044)
Supplement: S1 File — (DOCX) [file pone.0319044.s001.docx]

We utilized the following combined text and MeSH terms: "remimazolam" and "quality of recovery." The PubMed search strategy was as follows:

#1 "remimazolam"[Supplementary Concept] OR "ONO-2745"[Title/Abstract] OR "ONO2745"[Title/Abstract] OR "remimazolam"[Title/Abstract] OR "ONO-2745"[Title/Abstract] OR "cns 7056"[Title/Abstract];

#2 "randomized controlled trial"[Publication Type] OR "randomized"[Title/Abstract] OR "placebo"[Title/Abstract];

#3 #1 AND #2.

The search strategy for the Embase database was as follows:

#1 'remimazolam'/exp OR 'remimazolam'

#2 'randomized controlled trial'/exp OR 'randomized':ab,ti OR 'placebo':ab,ti

#3 #1 AND #2.

The search strategy for the Cochrane database was:

#1 - (remimazolam):ti,ab,kw.

For the Web of Science database, the search strategy was:

remimazolam (Abstract) AND randomized (Abstract).
