# Supplementary material for: Effect of intraoperative intravenous remimazolam on the postoperative quality of recovery after noncardiac surgery: A meta-analysis of randomized controlled trials
Source: PLoS One. 2025 Mar 20;20(3):e0319044. doi: 10.1371/journal.pone.0319044 (PMC11925310; doi:10.1371/journal.pone.0319044)
Supplement: S4 File — (DOCX) [file pone.0319044.s004.docx]

**Author(s):**
**Date:** 2024-10-19
**Question:** Should remimazolam be used for anethesia?
**Settings:**
**Bibliography:** . remimazolam for anesthesia. Cochrane Database of Systematic Reviews [Year], Issue [Issue].

| **Quality assessment** | | | | | | | **No of patients** | | **Effect** | | **Quality** | **Importance** |  |
| --- | --- | --- | --- | --- | --- | --- | --- | --- | --- | --- | --- | --- | --- |
|  |  |  |  |  |  |  |  |  |  |  |  |  |  |
| **No of studies** | **Design** | **Risk of bias** | **Inconsistency** | **Indirectness** | **Imprecision** | **Other considerations** | **Remimazolam** | **Control** | **Relative (95% CI)** | **Absolute** |  |  |  |
| **Postoperative quality of recovery (Better indicated by lower values)** | | | | | | | | | | | | |  |
| 11 | randomised trials | no serious risk of bias | serious^1^ | no serious indirectness | no serious imprecision | none | 632 | 638 | - | SMD 0.12 higher (0.13 lower to 0.36 higher) | ⊕⊕⊕O MODERATE | IMPORTANT |  |
| **Emotional status (Better indicated by lower values)** | | | | | | | | | | | | |  |
| 7 | randomised trials | no serious risk of bias | serious^1^ | no serious indirectness | no serious imprecision | none | 437 | 440 | - | SMD 0.16 higher (0.21 lower to 0.53 higher) | ⊕⊕⊕O MODERATE | IMPORTANT |  |
| **Physical comfort (Better indicated by lower values)** | | | | | | | | | | | | |  |
| 7 | randomised trials | no serious risk of bias | no serious inconsistency | no serious indirectness | no serious imprecision | none | 437 | 440 | - | SMD 0.13 higher (0.1 lower to 0.37 higher) | ⊕⊕⊕⊕ HIGH | IMPORTANT |  |
| **Psychological support (Better indicated by lower values)** | | | | | | | | | | | | |  |
| 7 | randomised trials | no serious risk of bias | serious^1^ | no serious indirectness | no serious imprecision | none | 437 | 423 | - | SMD 0.18 higher (0.14 lower to 0.51 higher) | ⊕⊕⊕O MODERATE | IMPORTANT |  |
| **Physical independence (Better indicated by lower values)** | | | | | | | | | | | | |  |
| 7 | randomised trials | no serious risk of bias | no serious inconsistency | no serious indirectness | no serious imprecision | none | 437 | 440 | - | SMD 0.05 higher (0.19 lower to 0.29 higher) | ⊕⊕⊕⊕ HIGH | IMPORTANT |  |
| **Pain (Better indicated by lower values)** | | | | | | | | | | | | |  |
| 7 | randomised trials | no serious risk of bias | no serious inconsistency | no serious indirectness | no serious imprecision | none | 439 | 440 | - | SMD 0.02 lower (0.22 lower to 0.19 higher) | ⊕⊕⊕⊕ HIGH | IMPORTANT |  |
| **VAS (Better indicated by lower values)** | | | | | | | | | | | | |  |
| 10 | randomised trials | no serious risk of bias | serious^1^ | no serious indirectness | no serious imprecision | none | 541 | 540 | - | MD 0.12 lower (0.41 lower to 0.17 higher) | ⊕⊕⊕O MODERATE | IMPORTANT |  |
| **MMSE (Better indicated by lower values)** | | | | | | | | | | | | |  |
| 3 | randomised trials | no serious risk of bias | no serious inconsistency | no serious indirectness | very serious^2^ | none | 102 | 103 | - | MD 0.95 higher (0.4 to 1.5 higher) | ⊕⊕OO LOW | IMPORTANT |  |
| **Duration of Pacu stay (Better indicated by lower values)** | | | | | | | | | | | | |  |
| 10 | randomised trials | no serious risk of bias | serious^1^ | no serious indirectness | no serious imprecision | none | 532 | 536 | - | MD 0.53 lower (3.08 lower to 2.02 higher) | ⊕⊕⊕O MODERATE | IMPORTANT |  |
| **Postoperative hospital stay duration (Better indicated by lower values)** | | | | | | | | | | | | |  |
| 6 | randomised trials | no serious risk of bias | serious^1^ | no serious indirectness | no serious imprecision | none | 278 | 278 | - | MD 0.06 lower (0.17 lower to 0.04 higher) | ⊕⊕⊕O MODERATE | IMPORTANT |  |
| **Time to extubation (Better indicated by lower values)** | | | | | | | | | | | | |  |
| 10 | randomised trials | no serious risk of bias | serious^1^ | no serious indirectness | no serious imprecision | none | 525 | 526 | - | MD 0.19 lower (3.68 lower to 3.29 higher) | ⊕⊕⊕O MODERATE | IMPORTANT |  |

^1^ The I2 is large.
^2^ Total population size is less than 400

| **remimazolam for anethesia** | | | | | | |
| --- | --- | --- | --- | --- | --- | --- |
| **Patient or population:** patients with anethesia **Settings:**  **Intervention:** remimazolam | | | | | | |
| **Outcomes** | **Illustrative comparative risks* (95% CI)** | | **Relative effect (95% CI)** | **No of Participants (studies)** | **Quality of the evidence (GRADE)** | **Comments** |
|  | Assumed risk | Corresponding risk |  |  |  |  |
|  | **Control** | **Remimazolam** |  |  |  |  |
| **Postoperative quality of recovery** |  | The mean postoperative quality of recovery in the intervention groups was **0.12 standard deviations higher** (0.13 lower to 0.36 higher) |  | 1270 (11 studies) | ⊕⊕⊕⊝ **moderate**^1^ | SMD 0.12 (-0.13 to 0.36) |
| **Emotional status** |  | The mean emotional status in the intervention groups was **0.16 standard deviations higher** (0.21 lower to 0.53 higher) |  | 877 (7 studies) | ⊕⊕⊕⊝ **moderate**^1^ | SMD 0.16 (-0.21 to 0.53) |
| **Physical comfort** |  | The mean physical comfort in the intervention groups was **0.13 standard deviations higher** (0.1 lower to 0.37 higher) |  | 877 (7 studies) | ⊕⊕⊕⊕ **high** | SMD 0.13 (-0.1 to 0.37) |
| **Psychological support** |  | The mean psychological support in the intervention groups was **0.18 standard deviations higher** (0.14 lower to 0.51 higher) |  | 860 (7 studies) | ⊕⊕⊕⊝ **moderate**^1^ | SMD 0.18 (-0.14 to 0.51) |
| **Physical independence** |  | The mean physical independence in the intervention groups was **0.05 standard deviations higher** (0.19 lower to 0.29 higher) |  | 877 (7 studies) | ⊕⊕⊕⊕ **high** | SMD 0.05 (-0.19 to 0.29) |
| **Pain** |  | The mean pain in the intervention groups was **0.02 standard deviations lower** (0.22 lower to 0.19 higher) |  | 879 (7 studies) | ⊕⊕⊕⊕ **high** | SMD -0.02 (-0.22 to 0.19) |
| **VAS** |  | The mean vas in the intervention groups was **0.12 lower** (0.41 lower to 0.17 higher) |  | 1081 (10 studies) | ⊕⊕⊕⊝ **moderate**^1^ |  |
| **MMSE** |  | The mean mmse in the intervention groups was **0.95 higher** (0.4 to 1.5 higher) |  | 205 (3 studies) | ⊕⊕⊝⊝ **low**^2^ |  |
| **Duration of Pacu stay** |  | The mean duration of pacu stay in the intervention groups was **0.53 lower** (3.08 lower to 2.02 higher) |  | 1068 (10 studies) | ⊕⊕⊕⊝ **moderate**^1^ |  |
| **Postoperative hospital stay duration** |  | The mean postoperative hospital stay duration in the intervention groups was **0.06 lower** (0.17 lower to 0.04 higher) |  | 556 (6 studies) | ⊕⊕⊕⊝ **moderate**^1^ |  |
| **Time to extubation** |  | The mean time to extubation in the intervention groups was **0.19 lower** (3.68 lower to 3.29 higher) |  | 1051 (10 studies) | ⊕⊕⊕⊝ **moderate**^1^ |  |
| *The basis for the **assumed risk** (e.g. the median control group risk across studies) is provided in footnotes. The **corresponding risk** (and its 95% confidence interval) is based on the assumed risk in the comparison group and the **relative effect** of the intervention (and its 95% CI).  **CI:** Confidence interval; | | | | | | |
| GRADE Working Group grades of evidence **High quality:** Further research is very unlikely to change our confidence in the estimate of effect.  **Moderate quality:** Further research is likely to have an important impact on our confidence in the estimate of effect and may change the estimate. **Low quality:** Further research is very likely to have an important impact on our confidence in the estimate of effect and is likely to change the estimate. **Very low quality:** We are very uncertain about the estimate. | | | | | | |
| ^1^ The I2 is large. ^2^ Total population size is less than 400 | | | | | | |
